# Complement C1q is hydroxylated by collagen prolyl 4 hydroxylase and is sensitive to off-target inhibition by prolyl hydroxylase domain inhibitors that stabilize hypoxia-inducible factor

**DOI:** 10.1016/j.kint.2017.03.008

**Published:** 2017-10

**Authors:** Serafim Kiriakidis, Simon S. Hoer, Natalie Burrows, Gloria Biddlecome, Moddasar N. Khan, Cyrille C. Thinnes, Christopher J. Schofield, Norma Rogers, Marina Botto, Ewa Paleolog, Patrick H. Maxwell

**Affiliations:** 1 Kennedy Institute of Rheumatology, NDORMS, University of Oxford, Oxford, UK; 2 School of Clinical Medicine, Cambridge Institute for Medical Research, Cambridge Biomedical Campus, University of Cambridge, Cambridge, UK; 3 Oncology Research, AMGEN, Thousand Oaks, California, USA; 4 Cobra Biologics, Keele, UK; 5 Department of Chemistry, University of Oxford, Oxford, UK; 6 Centre for Complement and Inflammation Research (CCIR), Imperial College London, London, UK

**Keywords:** complement C1q, hypoxia-inducible factor, prolyl hydroxylase inhibitors

## Abstract

Complement C1q is part of the C1 macromolecular complex that mediates the classical complement activation pathway: a major arm of innate immune defense. C1q is composed of A, B, and C chains that require post-translational prolyl 4-hydroxylation of their N-terminal collagen-like domain to enable the formation of the functional triple helical multimers. The prolyl 4-hydroxylase(s) that hydroxylate C1q have not previously been identified. Recognized prolyl 4-hydroxylases include collagen prolyl-4-hydroxylases (CP4H) and the more recently described prolyl hydroxylase domain (PHD) enzymes that act as oxygen sensors regulating hypoxia-inducible factor (HIF). We show that several small-molecule prolyl hydroxylase inhibitors that activate HIF also potently suppress C1q secretion by human macrophages. However, reducing oxygenation to a level that activates HIF does not compromise C1q hydroxylation. *In vitro* studies showed that a C1q A chain peptide is not a substrate for PHD2 but is a substrate for CP4H1. Circulating levels of C1q did not differ between wild-type mice or mice with genetic deficits in PHD enzymes, but were reduced by prolyl hydroxylase inhibitors. Thus, C1q is hydroxylated by CP4H, but not the structurally related PHD hydroxylases. Hence, reduction of C1q levels may be an important off-target side effect of small molecule PHD inhibitors developed as treatments for renal anemia.

In the 1960s, it was established that the assembly of collagen into its mature, triple helical form requires prolyl hydroxylation, which is the conversion of proline residues in procollagen to hydroxyproline. The enzymes responsible were subsequently identified as collagen prolyl-4-hydroxylases (CP4Hs),^1^, ^2^ which are members of the superfamily of 2-oxoglutarate (2-OG)–dependent dioxygenases that have an Fe(II) atom at the active site and require oxygen and 2-OG as cosubstrates.

Another important group of prolyl 4-hydroxylases has more recently been identified: prolyl hydroxylase domain (PHD) enzymes. These are structurally related to CP4Hs and belong to the superfamily of 2-OG−dependent dioxygenases. They act as key oxygen sensors in metazoans, controlling the master regulator hypoxia-inducible factor (HIF) through the hydroxylation of HIF-α subunits.^3^ Consistent with their role as oxygen sensors, the K_m_ for oxygen of the PHD enzymes is substantially higher than that of CP4Hs.^4^ PHDs are under intensive investigation as potential therapeutic targets to promote erythropoiesis and ameliorate ischemic injury.^5^, ^6^ Several companies have developed PHD inhibitors with structural similarity to 2-OG, and these are being tested in clinical trials. The extent to which these may have off-target effects on CP4Hs is largely unexplored.

Prolyl 4-hydroxylation also occurs in other proteins besides collagen and HIF-α subunits but has been less extensively investigated. One important example is the complement protein complex C1q.^7^, ^8^, ^9^ C1q comprises 18 polypeptide chains, assembled as 6 heterotrimers containing C1q A, B, and C chains, with an N-terminal triple helical collagen-like structure and a C-terminal globular region.^10^ C1q interacts with complement fixation sites of Igs to initiate complement activation and is mainly produced by bone marrow–derived cells.^11^, ^12^, ^13^ The importance of C1q is underlined by the fact that C1q deficiency causes susceptibility to infections and the autoimmune disease systemic lupus erythematosus.^14^

In this study, we aimed to determine whether PHD enzymes were responsible for the hydroxylation of C1q and whether small molecules developed as HIF activators may affect C1q.

## Results

### Prolyl hydroxylase inhibitors, but not hypoxia, reduce C1q secretion

**Figure 1:**
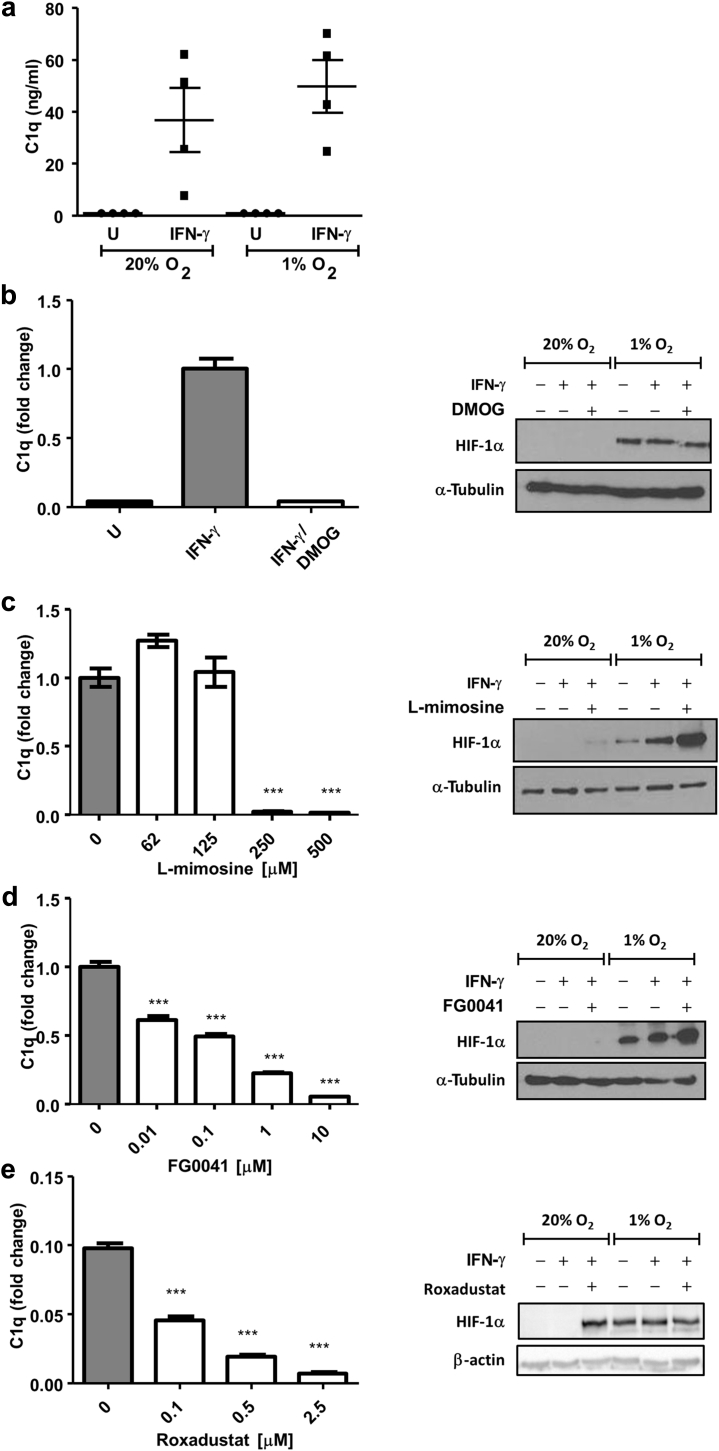
**C1q secretion by THP 1–derived macrophages (TDMs) is inhibited by prolyl hydroxylase domain inhibitors.** (**a**) TDMs were treated with interferon (IFN)-γ (10 ng/ml) or left untreated (U) and exposed to either normoxia (20% O_2_) or hypoxia (1% O_2_) for 16 hours. C1q secretion in the culture supernatants was measured using enzyme-linked immunosorbent assay (ELISA). Each point represents 1 experiment, and data are shown as means ± SEM. No significant differences were observed in IFN-γ–treated TDMs under normoxia versus hypoxia. (**b–e**) C1q production in TDMs was induced with IFN-γ (10 ng/ml) in the presence or absence of 16-hour treatment with dimethyloxalylglycine (DMOG; 62 μM, **b**), L-mimosine (250 μM, **c**), FG0041 (10 μM, **d**), or roxadustat (2.5 μM, **e**), as measured using ELISA. Protein levels relative to IFN-γ–treated controls are shown. Bar graphs show means ± SEM of 4–6 experiments. *** *P* < 0.001 versus IFN-γ alone. Hypoxia-inducible factor (HIF)-1α proteins were analyzed using Western blotting, with α-tubulin or β-actin used as the loading controls.

**Figure S1:**
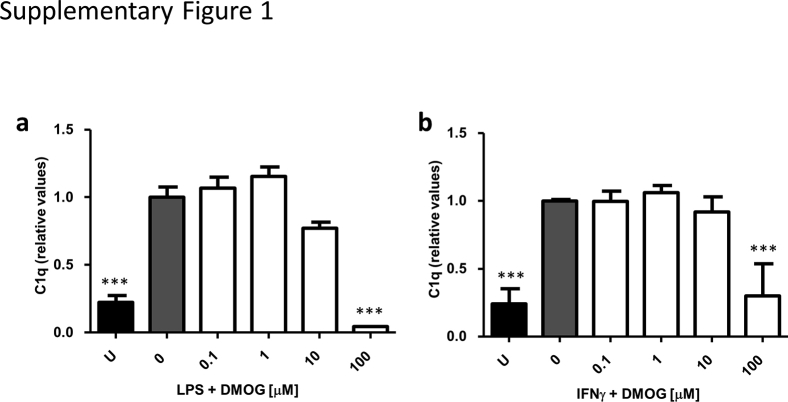
C1q expression in human monocyte-derived macrophages (MDMs) is inhibited by dimethyloxalylglycine (DMOG). MDMs were treated with (**A**) lipopolysaccharide (LPS, 10 ng/ml) or (**B**) interferon (IFN)-γ (10 ng/ml), with or without DMOG for 16 hours. C1q expression in culture supernatants was measured using enzyme-linked immunosorbent assay (ELISA). Histograms show the means ± SEM of 3 experiments. *** *P* < 0.001 versus cells treated with either LPS or IFN-γ.

Although it is established that active C1q requires prolyl hydroxylation,^7^, ^15^ the enzymes responsible have not been previously identified. This raised the possibility that PHD enzymes are involved in C1q hydroxylation, which would have important implications for pursuing PHDs as therapeutic targets. Macrophages are a major source of C1q. To investigate the potential role of PHDs in C1q secretion by human cells, we stimulated human THP-1–derived macrophages (TDMs) with interferon-γ under normoxic or hypoxic conditions. Interestingly, we found that exposure to 1% O_2_, a hypoxia level that results in substantial HIF stabilization by decreasing the activity of PHDs, did not significantly reduce the secretion of C1q into the culture medium relative to that found in cultures under normoxia (Figure 1a). Next, we investigated the effect of dimethyloxalylglycine (DMOG), a cell-permeable 2-OG analog that is widely used as an HIF activator.^16^ DMOG is a prodrug; its hydrolysis product N-oxalylglycine is a broad spectrum 2-OG oxygenase inhibitor, which competes with 2-OG for binding to both PHDs and CP4Hs. DMOG potently inhibited the secretion of C1q; notably, this was observed at a DMOG concentration that did not result in detectable HIF-1α stabilization in normoxia by Western blot (Figure 1b). We also examined primary monocyte-derived macrophages (MDMs) from human blood and observed that DMOG efficiently reduced C1q levels in response to interferon-γ or lipopolysaccharide (Supplementary Figure S1A and B).

We next examined 3 other small molecules that have been shown to inhibit PHD enzymes and stabilize HIF-1α: L-mimosine, an iron chelator,^17^, ^18^ and two compounds structurally related to N-oxalylglycine, roxadustat (FG4592)^19^, ^20^ and FG0041.^21^ All 3 molecules decreased C1q secretion by macrophages in a concentration-dependent manner, and C1q levels were significantly reduced at doses that were lower than (L-mimosine and FG0041) or similar (roxadustat) to those required to stabilize HIF-1α efficiently (Figure 1c–e).

### Collagen prolyl hydroxylases are required for C1q secretion

**Figure 2:**
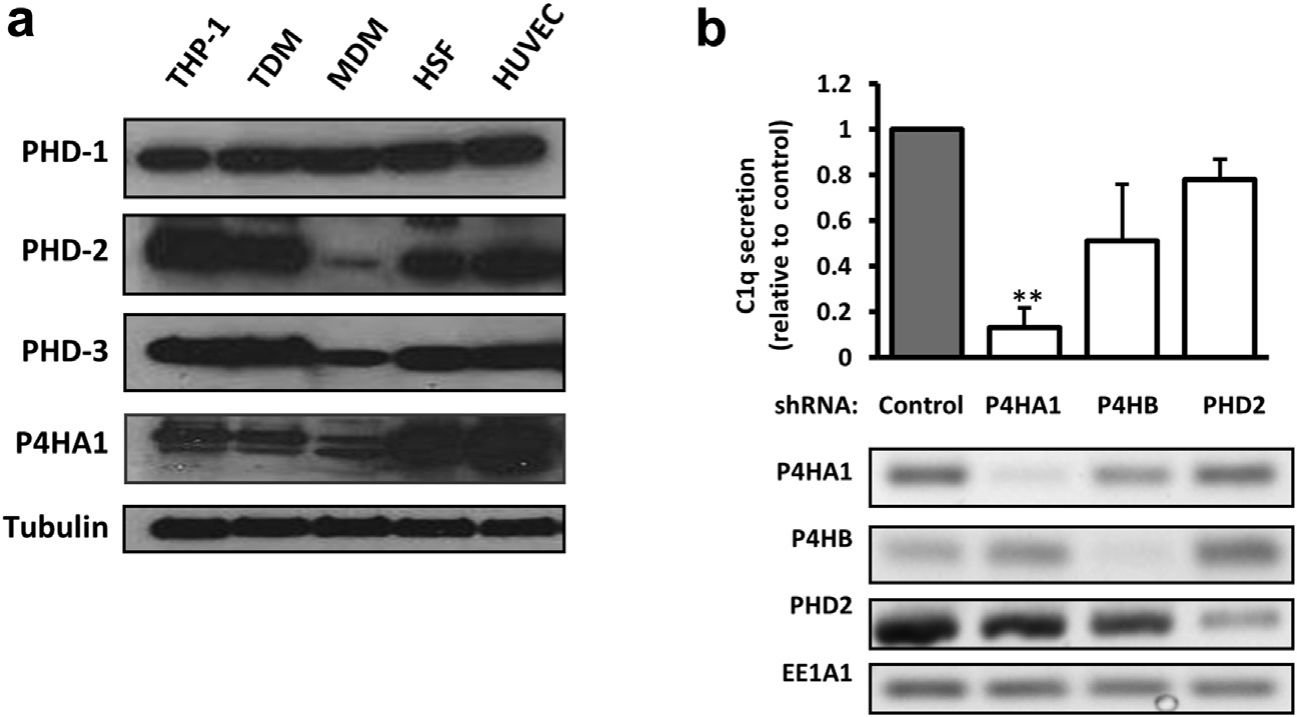
**C1q secretion is reduced after prolyl 4-hydroxylase alpha-1 (P4HA1) knockdown.** (**a**) Expression levels of prolyl hydroxylase domains (PHDs) in different cell types were examined using Western blotting, with α-tubulin used as the loading control. A blot representative of 3 independent experiments is shown. (**b**) Effect of knocking down the 2 subunits of collagen prolyl-4-hydroxylase 1 (CP4H1), P4HA and P4HB, or PHD2 in 293 cells stably transfected to express C1q. C1q expression in the culture supernatants was measured using enzyme-linked immunosorbent assay (ELISA). Bar graph shows means ± SEM of 3 experiments, expressed as fold changes over control: ** *P* < 0.01 versus control. Knockdown was confirmed by reverse transcription-polymerase chain reaction, with *EE1A1* transcript levels used as the control. EE1A1, Eukaryotic translation elongation factor 1 alpha 1; HSF, human skin fibroblasts; HUVEC, human umbilical vein endothelial cell; MDM monocyte-derived macrophage; shRNA, short hairpin RNA. To optimize viewing of this image, please see the online version of this article at www.kidney-international.org.

C1q secretion is relatively resistant to hypoxia and is sensitive to some small molecule prolyl hydroxylase inhibitors at doses below those required to stabilize HIF-1α. These findings implied that C1q hydroxylation was unlikely to be catalyzed by PHDs and that it may be mediated by CP4Hs. To further investigate this possibility, we examined whether the CP4H1-specific subunit prolyl 4-hydroxylase alpha-1 (P4HA1) was expressed in TDMs and MDMs and compared with those in fibroblasts, which are specialized collagen-producing cells. Protein analysis by Western blotting revealed that PHD1 to PHD3 and P4HA1 were expressed in all these cells (Figure 2a).

Although TDMs and MDMs are accepted models for studying C1q production, in our study, the observed secretion of C1q was modest. Furthermore, we reasoned that in this context, C1q production might be indirectly influenced, for example, through the activation of HIF that influences the cell-specific transcription of several hundred genes, including those encoding certain CP4H and PHD enzymes.^3^, ^22^, ^23^ To address these issues, we also examined the effect of decreasing PHD2 and P4HA1 levels in 293 cells engineered to produce recombinant C1q. Using lentiviral short hairpin RNA, we found that P4HA1 knockdown in these cells inhibited C1q secretion, whereas PHD2 knockdown did not (Figure 2b).

**Figure S2:**
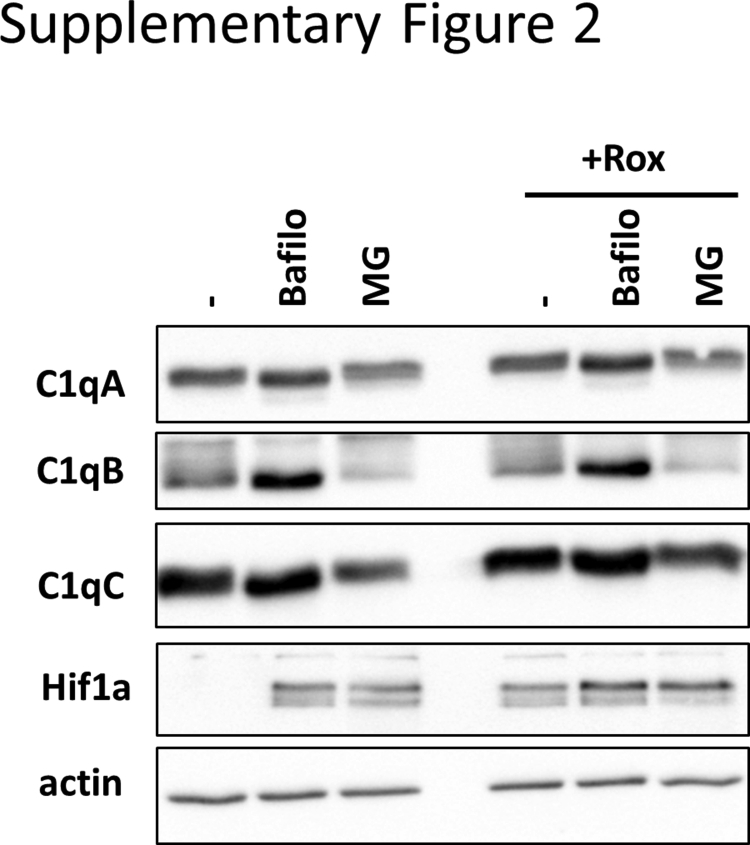
Recombinant C1q is degraded by the lysosomal pathway. Two hundred and ninety-three cells expressing recombinant C1q were treated with the lysosomal inhibitor bafilomycin A (Bafilo, 100 nM) or the proteasomal inhibitor MG132 (MG, 10 μg/ml) for 16 hours, with and without roxadustat treatment (Rox, 10 μM). Immunoblotting of cell lysates shows accumulation of C1q A, B, and C chains after bafilomycin treatment. Hypoxia-inducible factor (HIF)-1α, which was previously shown to be rescued by all 3 treatments (i.e., bafilomycin, MG132, and roxadustat) serves as the positive control, and β-actin serves as the loading control.

To explore the fate of C1q that was not assembled into a macromolecular complex and secreted, we examined the effect of inhibiting potential degradation pathways. We found that the lysosomal inhibitor bafilomycin substantially increased the amount of C1q in cell lysates, but the proteasomal inhibitor MG132 did not. This effect was observed even under standard culture conditions, likely reflecting imperfect stoichiometry of C1q components in the overexpression system (Supplementary Figure S2).

**Figure S3:**
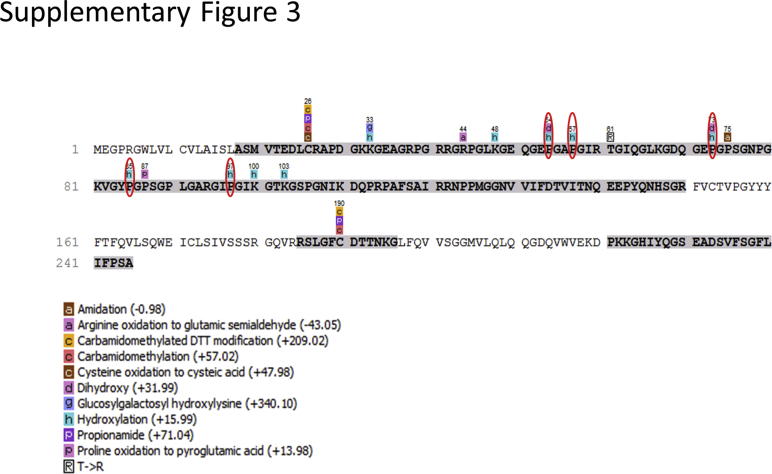
Posttranslational modification of recombinant C1q A chain. FLAG-tagged C1q protein was purified from the supernatants of 293 cells expressing all 3 C1q chains. The purified protein was separated using a polyacrylamide gel and digested in-gel using trypsin. The coverage is shown in gray highlights. The PEAKS software (Bioinformatics Solutions Inc.) was used to identify posttranslational modifications, as indicated above the modified amino acids. Hydroxylated proline residues are circled in red.

To examine the hydroxylation status of recombinant C1q secreted into the supernatants, we performed mass spectrometry analysis, which displayed a high degree of prolyl hydroxylation in collagen-like domains, consistent with previous reports for serum-derived C1q analyzed using amino acid sequencing (Supplementary Figure S3). To further characterize the manner in which roxadustat decreased the secretion of C1q, we examined intracellular C1q in the presence of bafilomycin to block degradation. We compared the hydroxylation status of intracellular C1q with and without roxadustat treatment using stable isotope labeling with amino acids in cell culture–based quantitative mass spectrometry. As predicted, a significant reduction of prolyl hydroxylation at multiple sites of intracellular C1q in treated samples was observed (Supplementary Table S1).

**Figure 3:**
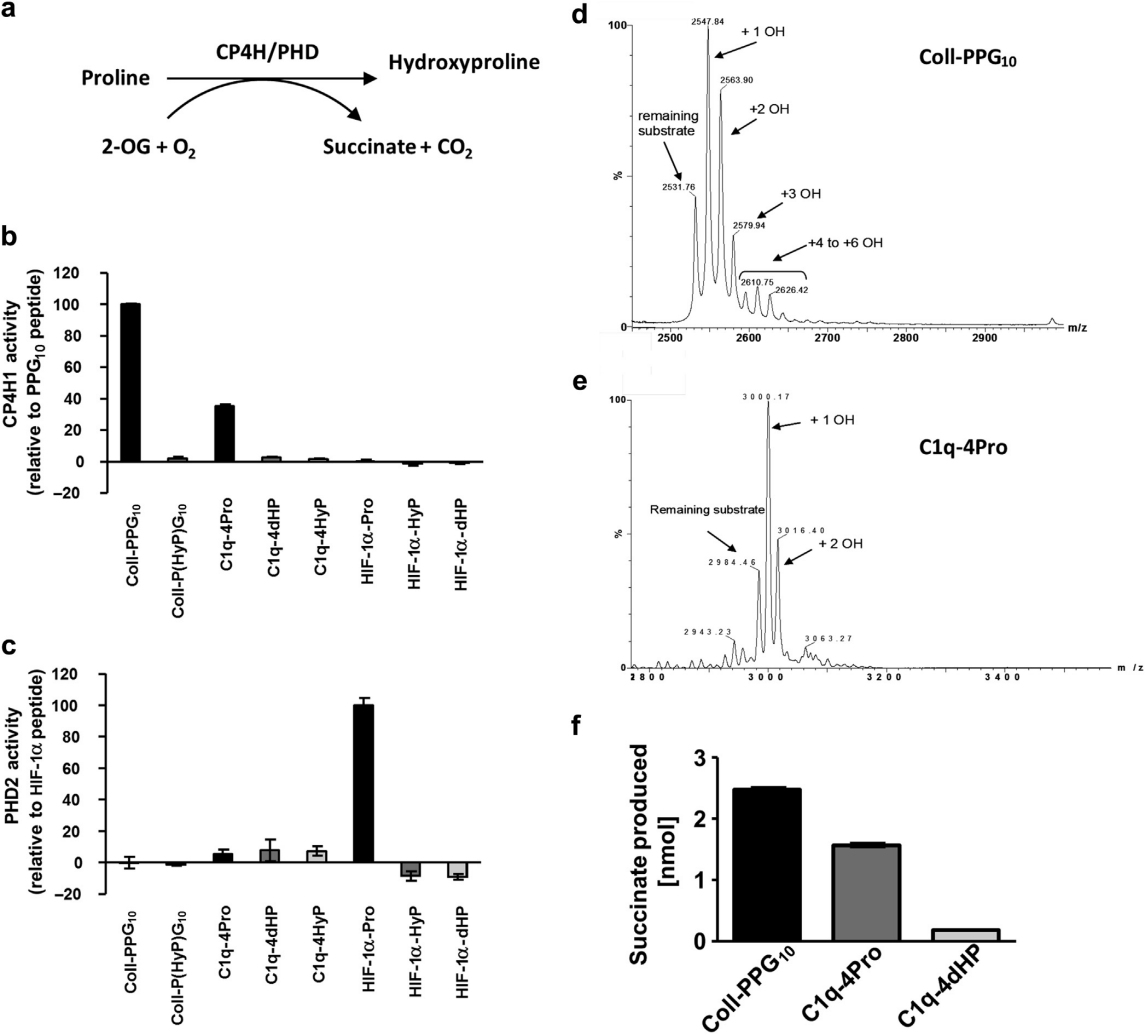
**C1q peptides are substrates for collagen prolyl-4-hydroxylase 1 (CP4H1) *in vitro.*** (**a**) Schematic depicting the role of CP4H and prolyl hydroxylase domain (PHD) enzymes in the hydroxylation of proline residues within target proteins and concomitant conversion of the essential cosubstrate 2-oxoglutarate (2-OG) to succinate. (**b–c**) Peptides derived from hypoxia-inducible factor (HIF)-1α, C1q A chain, or collagen (PPG_10_) were incubated with enzyme for 2.5 hours to stimulate 2-OG conversion to succinate. (**b**) Reactions contained 35 nM CP4H1 enzyme, 250 μM 2-OG, and 10 μM ferrous sulfate (FeSO_4_) and 50 μM peptides. C1q peptides that substituted all 4 prolines with 4-hydroxyproline (C1q-4HyP) or dehydroproline (C1q-4dHP) did not support CP4H1 enzyme activity, indicating that the observed reaction did not occur because of substrate uncoupled turnover of 2-OG and was specific to proline residues. (**c**) Reactions contained 120 nM PHD2, 20 μM 2-OG, and 100 μM FeSO_4._ Percent conversion of 2-OG to succinate was normalized to either PPG_10_ peptide (**b**) or HIF-1α peptide (**c**). Values are expressed as averages ± SD (n = 3 from a single experiment). Findings were reproduced in at least 1 additional experiment for each peptide and enzyme. (**d,e**) Mass spectrometry analysis of PPG_10_ and C1q-4Pro peptides following incubation with CP4H1 as described in (**b**) except nonradiolabeled 2-OG was used. (**d**) For PPG_10_, nonhydroxylated peptide (M + H = 2532), along with species showing 1, 2, 3, and 4–6 OH events (M + H = 16 per OH), are evident in the spectrum. (**e**) For C1q-4Pro peptide, nonhydroxylated peptide (M + H = 2985), along with species showing 1 and 2 OH events are evident in the spectrum. (**f**) Succinate produced in parallel reactions to those shown in panels (**d**) and (**e**), except radiolabeled 2-OG was used. Experiments contained 35 nM CP4H1, 10 μM Fe, 250 μM 2-OG, and 50 μM peptide. Reactions were incubated for 3 hours.

To directly test the ability of CP4H and PHD enzymes to hydroxylate C1q, we performed *in vitro* enzyme assays using peptides derived from HIF-1α, C1q A chain (C1q-4Pro), and procollagen [(Pro-Pro-Gly)_10_; PPG_10_] as templates, with recombinant preparations of PHD2 and CP4H1 (Figure 3a). The C1q peptide was a substrate for CP4H1, as evidenced by the increased conversion of the cosubstrate 2-OG to succinate (Figure 3b), but it was not a substrate for PHD2 (Figure 3c). Mass spectrometry analysis of the peptide substrates confirmed that CP4H1 was able to hydroxylate both PPG_10_ and C1q-4Pro peptide on multiple sites (Figure 3d and 3e, respectively). Control reactions simultaneously performed using mass spectrometry samples showed concomitant conversion of 2-OG to succinate (Figure 3f).

### Prolyl hydroxylase inhibitors reduce C1q plasma levels *in vivo*

**Figure 4:**
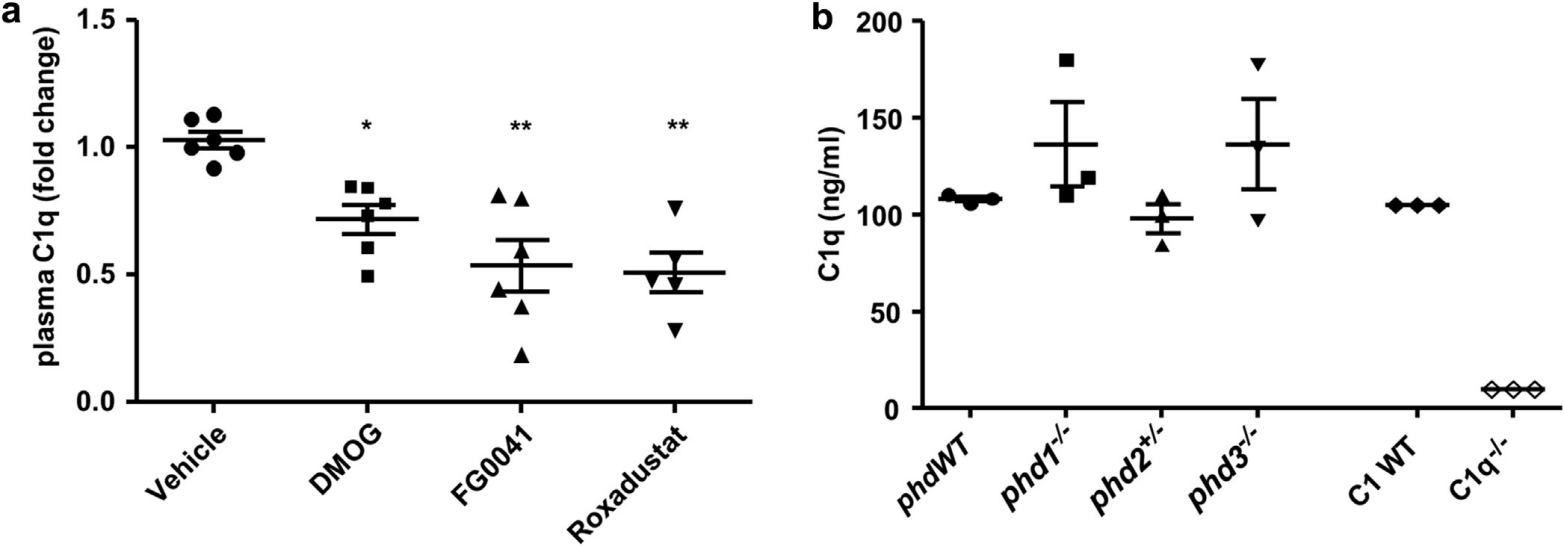
**Prolyl hydroxylase domain (PHD) inhibitors reduce circulating C1q in mice.** (**a**) C57BL/6 mice were treated every 12 hours with dimethyloxalylglycine (DMOG; 20 mg/kg), FG0041 (25 mg/kg), or roxadustat (10 mg/kg) for 6 days. Serum C1q levels were measured using enzyme-linked immunosorbent assay (ELISA). Blood was sampled prior to treatment (baseline) and 6 days after treatment. Changes in plasma C1q levels are expressed as fold changes from baseline. Plasma C1q levels were significantly reduced in mice treated with PHD inhibitors. Vehicle-treated mice were unaffected (* *P* < 0.05, ** *P* < 0.01 vs. baseline). Data represent means ± SEM of 5–6 mice per group. (**b**) C1q expression in mice with germline deletion of different PHD enzymes or C1q was measured by ELISA. Each symbol represents 1 mouse, with means ± SEM shown. WT, wild-type.

To establish whether the inhibitory effect of small molecule HIF activators on C1q secretion translated to the *in vivo* setting, C57BL/6 mice were treated with DMOG, FG0041, roxadustat, or vehicle every 12 hours for 6 days, and their effects on circulating C1q were assessed. The doses selected were similar to those reported to confer significant therapeutic effects in preclinical models of ischemic injury.^18^, ^20^, ^24^, ^25^ Plasma C1q was reduced by 28% with DMOG, 46% with FG0041, and 49% with roxadustat (Figure 4a). These data demonstrated the potential for small molecules that activate HIF to influence C1q and the complement pathway *in vivo* at therapeutically relevant doses. On the basis of the above evidence, this was likely an off-target effect, mediated via CP4H inhibition.

**Figure S4:**
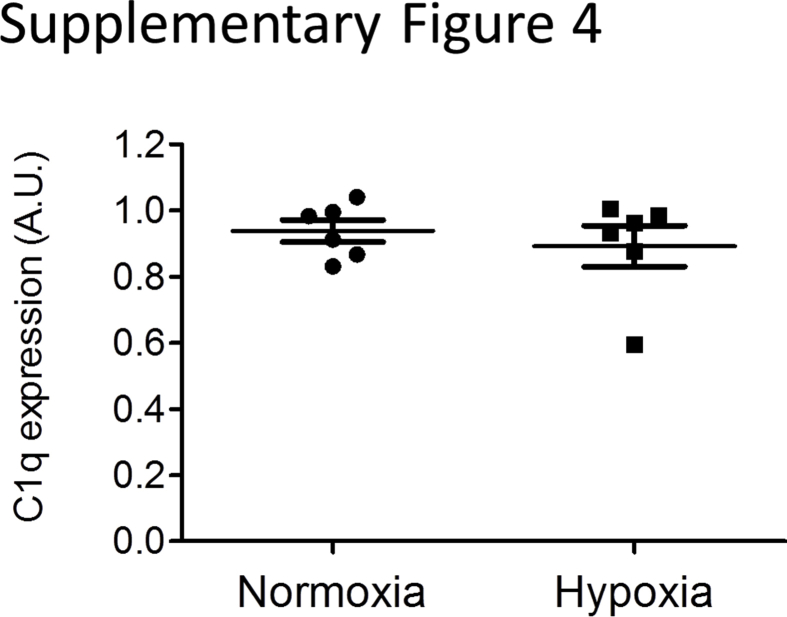
Mice exposed to hypoxia for 3 days exhibit no changes in circulating C1q levels. Mice (3 males and 3 females in each group, 70–77-day-old) were exposed to 10% O_2_ for 3 days. The serum C1q level at day 3 was measured with an in-house enzyme-linked immunosorbent assay (ELISA) and given as arbitrary units (AU).

To provide further evidence that inhibiting PHD enzymes *per se* was not sufficient to decrease C1q, we examined mice with genetic defects in PHD enzymes (*phd1*^*−/−*^, *phd2*^*+/−*^, and *phd*^*−/−*^) and observed that these mice did not show reduced circulating C1q levels (Figure 4b). Homozygous *phd2*^*−/−*^ mice were not viable; thus, we could not exclude the possibility that the residual PHD2 activity in heterozygotes influenced C1q secretion. Finally, we did not observe any significant changes in circulating C1q levels in mice exposed to hypoxia (10% O_2_) for 3 days (Supplementary Figure S4).

## Discussion

Together, our findings are consistent with the hydroxylation of C1q being mediated by CP4H and not by the PHD subfamily of prolyl hydroxylases. FG0041, DMOG and L-mimosine are all effective inhibitors of collagen synthesis,^26^ which is entirely consistent with our observation that they are effective inhibitors of C1q secretion and our conclusion that CP4H catalyzes C1q modification. Interestingly, *P4HA1* expression is increased in hypoxia via HIF-promoted transcription.^22^ This, together with the fact that CP4H enzymes have a lower K_m_ for oxygen than PHD enzymes,^4^ likely explains why C1q hydroxylation is maintained under hypoxic conditions (1% O_2_).

Our results revealed that CP4H1 was able to hydroxylate proline residues in the collagen domain of C1q, was expressed in macrophages, and was necessary for C1q secretion. We found that the 4 small molecules used to activate HIF also prevented C1q secretion by macrophages. We further showed that 2 inhibitors (DMOG and FG0041), which have been used in preclinical models, and one inhibitor (roxadustat, FG4592), which is currently in phase III clinical trials, significantly reduced circulating C1q levels in mice. These molecules are beneficial in models of ischemic disease, which has been attributed to HIF activation.^18^ Our findings further suggest that the inhibition of C1q secretion contributed to the observed benefit because C1q contributes to hypoxic-ischemic injury of several organs, including the brain and the heart,^27^, ^28^, ^29^ and the prevention of complement activation is considered a promising therapeutic strategy to limit ischemia-reperfusion injury to the kidneys.^30^, ^31^

In some circumstances such as ischemic injury, a multitargeted prolyl hydroxylase inhibitor that activates HIF and decreases C1q secretion might be attractive. In contrast, long-term reduction of C1q levels could occur when a nonselective prolyl hydroxylase inhibitor such as roxadustat is used to treat renal anemia. This might be harmful because genetic defects in C1q are associated with chronic infections and systemic lupus erythematosus.^14^ Similarly, Schindler *et al.* reported increased mortality in a sepsis model of kidney injury, possibly contributed to by off-target effects of the prolyl hydroxylase inhibitors used.^32^ Importantly, the fact that C1q secretion and HIF inactivation utilize structurally related oxygenases, which have different substrate selectivities and active sites, suggests that the development of PHD inhibitors that selectively activate HIF without compromising C1q secretion is possible.

## Materials and Methods

### Cell culture and reagents

THP-1 cells were obtained from ATCC, Maryland, USA. Human monocytes were obtained from single-donor plateletpheresis residues, as previously described.^33^ The 293 C1q cells were a kind gift from Nicole Thielens (IBS, France).^34^ DMOG was purchased from Frontier Scientific (Logan, UT). FG0041 was produced in-house.^21^ Roxadustat (FG4592) was purchased from ApexBio (Houston, TX). All other chemicals were obtained from Sigma Chemical Company (Poole, England, UK), unless otherwise indicated.

### Differentiation of monocytes into macrophages

TDMs were produced, as previously described.^35^ Human monocytes were differentiated into MDMs using macrophage colony-stimulating factor (PeproTech, London, UK) as previously described.^33^

### Stimulation of cells for C1q expression

C1q expression was induced in TDMs and MDMs by stimulation with interferon-γ (R&D systems, Minneapolis, MN) or lipopolysaccharide in Opti-MEM (Invitrogen, Paisley, UK) with or without PHD inhibitors. For hypoxic experiments, cells were exposed to 1% O_2_ using a hypoxic incubator (Galaxy R; Biotech, Palo Alto, CA).

### Enzyme-linked immunosorbent assay for C1q

Media were harvested after 48 hours and stored at −80°C. C1q was measured using a commercial human C1q enzyme-linked immunosorbent assay (ELISA; eBioscience, Hatfield, UK), according to the manufacturer’s instructions or an in-house sandwich ELISA. In brief, microtiter plates (Nunc-Immunoplate II, BRL, Middlesex, UK) were coated with 100 μl (1:1000) human sheep polyclonal anti-C1q (The Binding Site Group Ltd, Birmingham, UK) in phosphate-buffered saline (PBS) by incubating overnight at 4°C. Plates were then washed twice with PBS containing 0.075% Tween 20, followed by 2 more washes with PBS only, and were then blocked with 200 μl PBS-2% bovine serum albumin (BSA) for 2 hours at 37°C. Mouse monoclonal anti-C1q (clone 1A4, Santa Cruz Biotechnology, Santa Cruz, CA) at 1:1000 dilution in PBS-2% BSA was then used as the detection antibody, followed by alkaline phosphatase–conjugated anti-mouse IgG (Sigma-Aldrich, Poole, UK) at 1:1000 in PBS-2% BSA. The substrate p-nitrophenyl phosphate (Sigma-Aldrich) dissolved in Tris-buffer was added to the plate and incubated for 30 minutes at room temperature in the dark. The plates ware read at 405 nm on a spectrophotometric ELISA plate reader.

Mouse plasma samples were analyzed using an in-house mouse C1q ELISA adapted from Petry *et al.*^13^ Microtiter plates (MaxiSorb; Nunc, Germany) were coated with 1 μg/ml C1q antibody (clone 7H8; Hycult Biotech, The Netherlands) and incubated overnight at 4°C. The wells were blocked with 1% BSA in PBS-0.05% Tween 20 for 1 hour at room temperature and then washed thrice with PBS-0.05% Tween 20 and once with PBS. Next, 100 μl of samples (diluted at 1:400 and 1:800) were added to wells and incubated for 1.5 hours at room temperature, washed as before, and incubated with 100 μl biotinylated C1q antibody (clone JL-1; Hycult Biotech) diluted at 1:4000 in PBS for 1 hour at room temperature. The wells were washed, as previously described, and incubated with 100 μl horseradish peroxidase–conjugated streptavidin (Biolegend, UK) diluted at 1:2000 in PBS for 1 hour at room temperature. The wells were washed and incubated with 100 μl 3,3′,5,5′-tetramethylbenzidine substrate solution for 10 to 20 minutes, and the reaction was stopped with 0.5 M H_2_SO_4_ (50 μl/well). The optical density was read at 450 nm (with the wavelength correction set at 540 and 570 nm).

### Stable prolyl hydroxylase knockdown

MISSION short hairpin RNA constructs for P4HA1 (TRCN0000303934), P4HB (TRCN000 O_2_96675), and PHD2 (TRCN0000001043) were purchased from Sigma-Aldrich. Lentiviral particles were produced in 293T cells by cotransfection of the short hairpin RNA vector and the packaging vectors pMD2.G and pCMV-dR8.2 (Addgene, Cambridge, MA) using TransIT-293 transfection reagent (Mirus Bio, Madison, WI), according to the manufacturer’s instructions. Filtered cell culture supernatants were added to 293 cells stably expressing C1q, followed by selection with puromycin. Stable knockdown was confirmed by reverse transcription-polymerase chain reaction using TRIzol-extracted RNA and SuperScript III reverse transcriptase (Invitrogen, Carlsbad, CA) according to the manufacturer’s instructions.

### Reverse transcription-polymerase chain reaction primer sequences 5′-3′

*P4HA1F1* AGACCTAGCAAAACCAAGGCT

*P4HA1R1* TTTCATAGCCAGAGAGCCAGG

*P4HBF2* CTTCAAGGACGTGGAGTCGG

*P4HBR2* ACCCCATCTTTGTCGAGCTG

*PHD2F2* ACTGGGATGCCAAGGTAAGTG

*PHD2R2* CTCGTGCTCTCTCATCTGCAT

*EE1A1F* AAGTGCTAACATGCCTTGGTTC

EE1A1R AGGAACAGTACCAATACCACCA

### Immunoblotting

Tissues and cells were homogenized in protein extraction buffer, and protein analysis was performed, as previously described.^36^ The following antibodies were used: mouse monoclonal anti-HIF-1α (clone 54; Transduction Labs, Lexington, KY), rabbit polyclonal anti-HIF-1α (NB100-479; Novus Biologicals, Littleton, CO), rabbit polyclonal anti-PHD-1, rabbit polyclonal anti-PHD-2, and goat polyclonal anti-P4HA1 (Abcam, Cambridge, MA). Alkaline phosphatase–conjugated anti-mouse IgG (γ chain specific), horseradish peroxidase–conjugated donkey anti-mouse and anti-rabbit IgG antibodies (Bethyl Laboratories, Montgomery, TX), and mouse monoclonal anti-human α-tubulin and β-actin antibodies were purchased from Sigma-Aldrich.

### PHD2 and CP4H1 enzymatic assays

The CP4H1 and PHD2 enzyme assays measured the conversion of 5-[^14^C]-2-oxoglutarate to [^14^C]-succinic acid. For CP4H1 reactions, final conditions included 50 mM Tris-HCl pH 7.5, 0.2 mM dithiothreitol, 1 mM ascorbate, 4% dimethylsulfoxide, and 0.5 mg/ml catalase. For PHD2 reactions, final conditions included 30 mM 2-(N-morpholino) ethanesulfonic acid pH 6.0, 10 mM NaCl, 5 mM CaCl_2_, 2.5 mM dithiothreitol, 0.25% Brij-35, 0.05 mg/ml BSA, 4% dimethylsulfoxide, and 2 mM ascorbate. Concentrations of FeSO_4_, enzyme, peptide substrates, and 2-OG are shown in the legend of Figure 2. In brief, FeSO_4_, enzyme, and peptide were sequentially added to the assay buffer and gently mixed for 10 minutes at room temperature. 2-OG was then added to initiate the reaction. Reactions were gently mixed at room temperature and terminated after 3 or 3.5 hours by adding an equal volume of 0.02 N H_2_SO_4_. A portion of each terminated reaction was injected into a Polypore H column (PerkinElmer, Waltham, MA) at a rate of 0.3 ml/min with 0.01 N H_2_SO_4_ as the mobile phase. Substrate and product peaks were detected at 210 nm (Agilent 1100, Agilent Technologies, Santa Clara, CA), and the radioactivity associated with each was captured using a Beta-RAM Model 2 radiation detector and the In-Flow 2:1 scintillation cocktail (IN/US Systems Inc., Tampa, FL). Laura Lite software (IN/US Systems) was used to collect and analyze radiometric data. This high-performance liquid chromatography method exploits the difference in pKa of 2-OG and succinic acid carboxylates to chromatographically separate substrate from product at low pH using ion exchange resin, as described by Cunliffe *et al.* and Kaule and Günzler.^37^, ^38^

The sequence of C1q A chain peptides with C-terminal amides (C1q-4 Pro) was H-GEAGR[Pro]GRRGR[Pro] GLKGEQGE[Pro][GA[Pro]GIRTGI-OH. For C1q-4 dHP and C1q-4 HyP, the proline motifs were substituted with dehydroproline and 4-hydroxyproline, respectively. The sequence selected for testing was based on the findings of Reid.^8^ The sequence of HIF-1α peptides (HIF-1α Pro) was H-DLDLEMLA[Pro] YIPMDDD-FQL-OH. For HIF-1α dHP and HIF-1α HyP, the proline motifs were substituted with dehydroproline and 4-hydroxyproline, respectively.

The collagen peptide PPG_10_ was purchased from Peptides International (Louisville, KY).

The following materials were obtained from their respective manufacturers: 5-[^14^C]-2-oxoglutarate (2-OG), PerkinElmer LAS (Shelton, CT) or Moravek Biochemicals (Brea, CA); complement C1q peptides, MidWest Biotech (Fishers, IN); and catalase (Sigma-Aldrich). HIF peptides were internally generated (Amgen) or obtained from MidWest Biotech.

### Mass spectrometry of peptides

Reactions with unlabeled 2-OG were run in parallel with reactions containing radiolabeled 2-OG and allowed to proceed for 3 hours. Reactions containing unlabeled 2-OG were prepared for matrix-assisted laser desorption/ionization-mass spectrometry as follows: 1 μl sample was diluted to 10 μl α-cyano-4-hydroxycinnamic acid (10 mg/ml in 50% acetonitrile containing 0.05% trifluoroacetic acid), and 1 μl of the resultant solution was air dried on stainless steel. The sample mass was measured using a Waters MALDI Micro MX mass spectrometer (Waters, Milford, MA). The reflectron mode (using an acceleration voltage of 25 kV) in combination with delayed extraction was employed. The mass spectrometer was externally calibrated using a mixture of neurotensin, angiotensin, and 3 adrenocorticotropic hormone fragments (1–17, 18–39, and 7–38). The ^14^C-labeled control reactions were analyzed by high-performance liquid chromatography, as described above.

### Stable isotope labeling with amino acids in cell culture and mass spectrometry

Cells were labeled with heavy or light arginine and lysine for at least 5 passages. Cells were treated with roxadustat or vehicle (dimethylsulfoxide) and bafilomycin A to inhibit lysosomal acidification and prevent any loss of nonhydroxylated protein marked for degradation. After 20 hours, roxadustat-treated cells were combined with controls and lysed in 1% Triton X-100 before C1q protein was purified by FLAG pull-down and separated using a polyacrylamide gel. Samples were reduced, alkylated, and digested in-gel using trypsin, with the resulting peptides eluted for analysis using liquid chromatography-tandem mass spectrometry. Peptides were analyzed using a Q Exactive mass spectrometer (Thermo Fisher Scientific, Waltham, MA) coupled to an RSLC nano3000 UPLC (Thermo Fisher Scientific). Data were acquired in a DDA fashion. Raw files were processed in PEAKS Studio 8.0 (Bioinformatics Solutions Inc., Waterloo, ON, Canada) or Proteome Discoverer 1.4 software (Thermo Fisher Scientific). Data were searched using the Uniprot database (465,339 sequences, downloaded 11/07/16) and the human Uniprot database (30,510 sequences, downloaded 06/19/16). Using the Proteome Discoverer software, variable modifications were set as 13C(6)15H(2) lysine, 13C(6)15N(4) arginine, and oxidized methionine and proline. The PEAKS software was used to search for posttranslational modifications, with confident modification sites requiring a minimum ion intensity of 5%.

### *In vivo* studies

All procedures were ethically approved and complied with the Scientific Procedures Act 1986 and the Guidelines for the Welfare and Use of Animals in Cancer 2010,^39^ under the authority of the Home Office, UK. Mice with defective *phd* and *c1q* alleles were previously described.^40^, ^41^ For inhibitor studies, male and female C57BL/6 mice (aged 12 weeks) were randomly assigned to treatment groups (4 groups, 5–6/group; calculated using the resource equation). Prior to treatment, blood was sampled for analyzing baseline plasma C1q levels. Mice were then treated with 20 mg/kg DMOG, 25 mg/kg FG0041, 10 mg/kg roxadustat, or vehicle (4% dimethylsulfoxide in PBS) and dosed with 0.1 ml/10 g body weight with an i.p. injection every 12 hours for 6 days. A second blood sample was collected for analyzing plasma C1q after killing the mice.

### Statistical analysis

All data were analyzed by 1-way analysis of variance with Bonferroni or Tukey *post hoc* correction.

## Disclosures

PHM and CJS are scientific founders and equity holders of ReOx, which aims to develop PHD inhibitors as therapies. All the other authors declared no competing interests.

## Supplementary Material

Table S1 Reduced prolyl hydroxylation of C1q A chain after roxadustat treatment.
