# Supplementary material for: Complement C1q is hydroxylated by collagen prolyl 4 hydroxylase and is sensitive to off-target inhibition by prolyl hydroxylase domain inhibitors that stabilize hypoxia-inducible factor
Source: Kidney Int. 2017 Oct;92(4):900–8. doi: 10.1016/j.kint.2017.03.008 (PMC5612014; doi:10.1016/j.kint.2017.03.008)
Supplement: Table S1 — Reduced prolyl hydroxylation of C1q A chain after roxadustat treatment. [file mmc1.docx]

**Supplementary Table S1**

**Reduced prolylhydroxylation of C1qA after roxadustat treatment.**

| C1qA peptide | PTM | Rox/contr. | Contr/Rox |
| --- | --- | --- | --- |
| **VGYPGpSGPLGAR** | **P6(Oxidation)** | **0.161** | **2.546** |
| VGYPGPSGPLGAR |  | 0.797 | 0.874 |
| **GRPGLKGEQGEPGApGIR** | **P15(Oxidation)** | **0.165** | **2.421** |
| GRPGLKGEQGEPGAPGIR |  | 1.023 | 1.199 |
| TGIQGLKGDQGEpGPSGNPGKVGYPGPSGPLGAR | P13(Oxidation) | 0.373 | 0.608 |
| TGIQGLKGDQGEPGPSGNPGKVGYPGPSGPLGAR |  | 0.789 | - |
| TGIQGLkGDQGEpGPSGNPGk | P13(Oxidation) | 0.860 | 0.933 |
| TGIQGLKGDQGEPGPSGNPGK |  | 1.108 | 1.114 |
| GDQGEPGPSGNPGK |  | 0.973 | 1.058 |
| DQPRPAFSAIR |  | 1.000 | 1.454 |
| GSPGNIKDQPRPAFSAIR |  | 1.011 | 1.240 |
| GEQGEPGAPGIR |  | 1.210 | 1.423 |

293 cells expressing C1q were SILAC labelled and treated with roxadustat or vehicle for 16 hours. Lysosomal degradation was blocked with bafilomycin A. FLAG-tagged C1qA was purified from cell lysates and analysed by mass spectrometry for changes in the hydroxylation status. The ratio of peptides derived from roxadustat (Rox) treated heavy labelled cells was strongly reduced for two of the hydroxylated peptides (in bold) compared to light labelled mock treated control cells (Rox/contr). The reverse labelling confirmed the low hydroxylation status of these two peptides (contr/Rox). Note that the ratio of the non-hydroxylated version does not significantly change. The post translational modification (PTM) hydroxylation is indicated as oxidation and the position within the peptide given.
